# Retroauricular Approach for Targeted Cochlear Therapy Experiments in Wistar Albino Rats

**DOI:** 10.4274/balkanmedj.2016.0226

**Published:** 2017-05-15

**Authors:** Selçuk Mülazımoğlu, Emre Ocak, Gülşah Kaygusuz, Mustafa Kürşat Gökcan

**Affiliations:** 1 Department of Otorhinolaryngology, Ankara University School of Medicine, Ankara, Turkey; 2 Department of Otorhinolaryngology, Keçiören Training and Research Hospital, Ankara, Turkey; 3 Department of Pathology, Ankara University School of Medicine, Ankara, Turkey

**Keywords:** Wistar albino rats, inner ear, Anatomy, operative surgical procedure

## Abstract

**Background:**

As the idea of stem cell technology in the treatment of sensorial hearing loss has emerged over the past decades, the need for in vivo models for related experiments has become explicit. One of the most common experimental models for inner ear stem cell delivery experiments is the Wistar albino rat.

**Aims:**

To investigate the surgical anatomy of the temporal bone of the Wistar albino rat with respect to the dissection steps, operative techniques and potential pitfalls of surgery.

**Study Design:**

Animal experimentation.

**Methods:**

Adult Wistar albino rats were operated on via the retroauricular approach under an operation microscope. The anatomy of the temporal bone, the surgical route to the temporal bulla and the inner ear were investigated. Technical details of surgical steps, complications and potential pitfalls during the surgery were noted.

**Results:**

The study group consisted of 40 adult Wistar albino rats. The mean times to reach the bulla and to achieve cochleostomy were 4.3 (2-13 min) and 7.5 min (3.5-22 min), respectively. The mean width of the facial nerve was 0.84 mm (0.42-1.25 mm). The stapedial artery lay nearly perpendicular to the course of the facial nerve (88-93 °C). There were three major complications: two large cochleostomies and one massive bleed from the stapedial artery.

**Conclusion:**

The facial nerve was the key anatomical landmark in locating the bulla. By retrograde tracing of the facial nerve, it was possible to find the bulla ventral (inferior) to the main trunk. The facial nerve trunk was the upper limit when drilling the bulla. By dissecting the main trunk of the facial nerve and retracting cranially, a large drilling space could be achieved. Our results suggest that the retroauricular approach is an effective, feasible route for inner ear drug delivery experiments in Wistar albino rats.

The treatment of sensorineural hearing loss stepped into a new age as stem cell studies for hairy cell restoration became popular. Deafened animals are frequently used as models for gene therapies (1). Investigations usually focus on the cell lines and the mediators; however, delivering the agent with the proper technique into the cochlea is as crucial as the agent itself (2,3). Scientists have been studying the inner ear in animal models for sensorineural hearing loss, yet the surgical anatomy of the temporal bones of experimental animals and effective surgical methods to reach the inner ear have not been well established. Guinea pigs, rats and mice are the most commonly used animals for otologic research, but a limited number of studies have been published so far (1,4,5).

The laboratory rat has been used extensively in auditory research, but has had limited use in targeted cochlear delivery-related research, mainly due to surgically restricted access to the cochlea and concomitant surgical considerations (2). However, the use of rats offers several experimental advantages over the use of other species given the low cost of this species, low mortality rate following surgical intervention, the extensive auditory research already performed and the applicability for molecular biological/proteomics research (2). Anatomical knowledge of the middle and inner ear of rats is vital to this type of research. Description of the surgical anatomy of the middle ear of rats was published previously (5,6,7). However, there is a paucity of anatomical dissection studies designed to guide researchers who wish to use the rat model for targeted inner ear therapy studies.

Our group conducted a series of targeted cochlear therapy experiments in Wistar albino rats (WARs), and we realized the paucity of data in the literature describing anatomical details in temporal bone dissection and cochleostomy procedures. Therefore, in this study, we combined our experience in dissecting the WAR temporal bone with respect to the dissection steps, operative techniques and potential pitfalls of the surgery. Visualization of the otic bulla, the basal turn of the cochlea and the hemicochlea preparation for histopathological analyses were emphasized.

## MATERIALS AND METHODS

We collected the data from temporal bone dissections of 40 adult female WARs. This study was approved by the Institutional Review Board (IRB) of the Ankara University Animal Studies Ethics Committee (IRB 2013-4-24). Animal care was in accordance with the Committee on Ankara University Laboratory Animal Care guidelines.

Animals were obtained from the experimental animal laboratory of Ankara University. Operations were performed on the right sides of the rats under a Leica M400 E surgical microscope (Leica, Germany). All the surgical dissection videos and notes were evaluated to reveal the surgical steps and anatomical structures as well as the times to reach the bulla and to achieve appropriate cochleostomy. Area measurements and geometric relations were calculated using image analysis software (NIH; Bethesda, MD, USA) from the static video frames.

### Temporal bone dissection

Rats were anesthetized with ketamine [4 mg/100 g, intramuscularly (IM)] and xylazine (1 mg/100 g, IM). All animals had preoperative prophylactic antibiotic protection (cephazoline 500 mg/kg/day, IM) (8,9). Rats were then placed in a lateral recumbent position. The postauricular region was shaved and sterilized with 10% povidone iodine. The rats were then draped with a 20x20 cm sterile surgical towel with a small hole placed over the surgical site.

**Figure 1:**
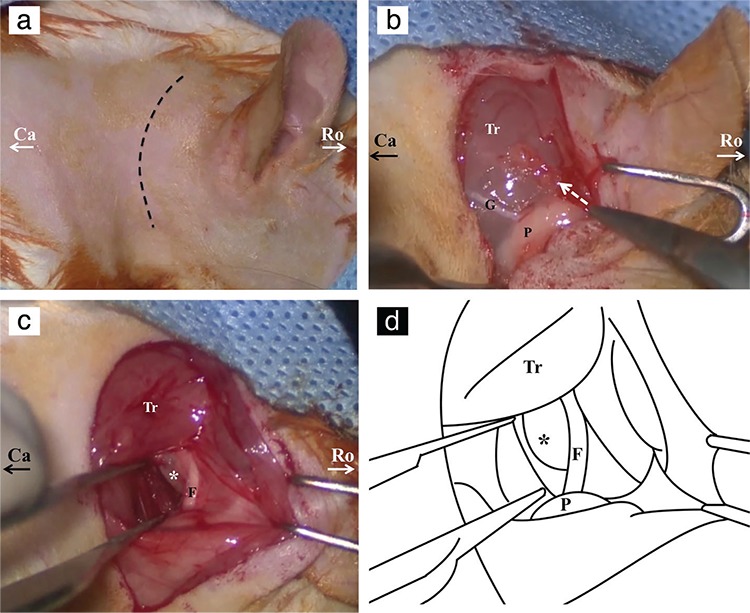
Dissection of otic bulla through retroauricular approach. Retroauricular incision of right ear (a). Dissection pathway after skin incision (dotted arrow) (b). Otic bulla (asterisk) and facial nerve of right ear (c). Schematic drawing showing otic bulla and facial nerve (d). *Ro: rostral side; Ca: caudal side; Tr: trapezius muscle; G: great auricular nerve; P: parotid gland; asterisk: otic bulla; F: facial nerve.*

**Figure 2:**
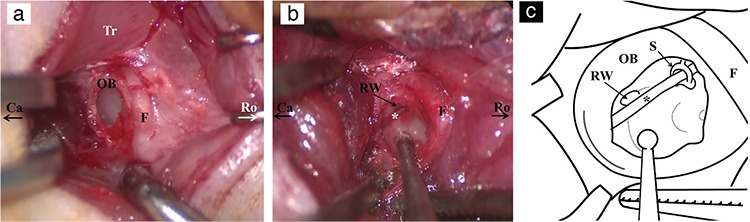
Opening of otic bulla and cochleostomy. Opening of otic bulla of right ear (a). Cochleostomy location according to stapedial artery (asterisk) (b). Schematic drawing of internal structures of otic bulla (c). *OB: otic bulla; Ro: rostral side; Ca: caudal side; F: facial nerve; Tr: trapezius muscle; RW: round window; S: stapes.*

The dissection was performed via the retroauricular approach. The following positional descriptions are used for rodent nomenclature and, therefore, are used in the manuscript: rostral refers to towards the head while caudal refers to towards the tail; ventral refers to the front while dorsal refers to the back (10). After making an approximately 3 cm retroauricular skin incision (Figure 1a), the auricle was gently pulled rostrally and the trapezius muscle, great auricular nerve and parotid gland were identified under the thin layer of platysma muscle (Figure 1b). The angle of the mandible could be palpated ventrally and rostrally to the parotid gland. The trapezius muscle was dissected and pulled caudally from its rostral border, and the main trunk of the facial nerve (FN) was identified (Figure 1c, 1d). Care was taken while dissecting the trapezius muscle and dissection proceeded dorsally to the great auricular nerve since ventral dissection risks damage to major blood vessels. By retrograde tracing of the FN, it was possible to locate the bulla ventral (inferior) to the main trunk. The periosteum of the bulla was removed with gentle manipulation by a no. 15 lancet [see video, supplemental digital content 1, which demonstrates the following: a. retroauricular incision of the right ear, b. dissection pathway after the skin incision (dotted arrow), c. identification and retrograde tracing of the FN and d. removal of the periosteum of the bulla; Tr: trapezius muscle, G: great auricular nerve, P: parotid gland]. Before the drilling stage, the bulla was free of periosteum and surrounding soft tissue. The cortical bone of the bulla was removed with a 3 mm diamond burr (Figure 2a). Because of the narrow surgical field, the shaft of the drill was prevented from contact with surrounding soft tissues. Angled bayonet forceps were used to provide a wide exposure area while drilling the bulla.

### Cochleostomy and injection

After the bulla was opened, the basal turn of the cochlea, round window (RW), ossicles and entire tympanic cavity could be visualized with gentle manipulation of the operation microscope and the rat. The stapedial artery was positioned at the caudal aspect of the cochlea (Figure 2b, 2c). The basal turn of the cochlea was drilled with a 1 mm diamond burr until it was thin enough to penetrate with a 30-gauge perforator to perform the cochleostomy (video 2c, see video, supplemental digital content 2, demonstrating a. drilling of the bulla, b. drilling of the cochlea and c. performing cochleostomy with a 30-gauge perforator; otic bulla, FN, asterisk: stapedial artery). We used a 30-gauge micro syringe (Hamilton Co., Bonaduz, Switzerland) with a micromanipulator for the injection. A small piece of muscle tissue was placed over the cochleostomy and fibrin sealant was applied briefly. The skin was closed with a 4.0 propylene suture.

### Cochlea extraction and hemicochlea preparation

**Figure 3:**
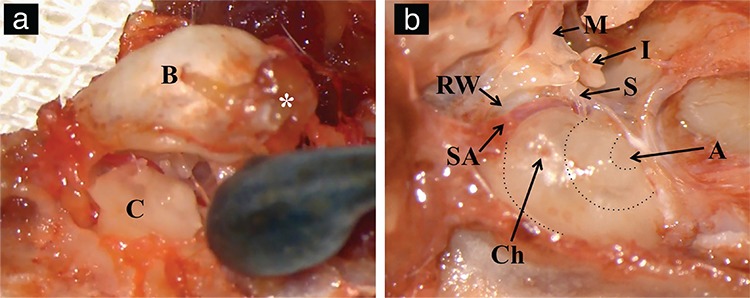
Extraction of cochlea. Detachment of bulla from rostral medial edge (asterisk) (a). Middle ear structures critical for cochleostomy (b). *B: right bulla, asterisk: rostral medial edge of the bulla, C: cochlea, SA: stapedial artery, Ch: cochleostomy site, dotted circles: turns of the cochlea, A: apex of the cochlea, RW: round window, S: stapes, I: incus, M: malleus.*

**Figure 4:**
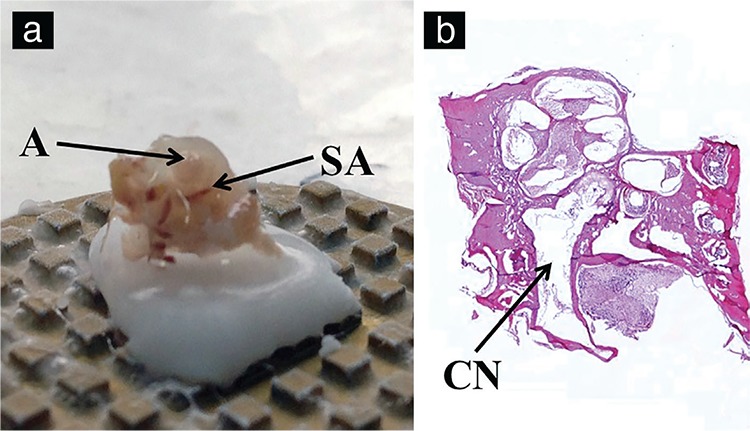
Cochlea extraction and hemicochlea preparation. Alignment of decalcified cochlea for sectioning (a). Histological preparation of cochlea H&E (x50) (b). *A: apex of the cochlea, SA: stapedial artery, CN: cochlear nerve.*

After sacrificing and decapitating the WARs, the mandibles were detached. The heads were sectioned in half coronally from the basispheniod and sagittally from the midline, to expose adjacent structures of temporal bone. Temporal bullas were detached from the suture lines with a Freer Elevator to get a better view of the middle ear cavity (Figure 3a). The rats were found to have a fragile junction of the tympanic bulla with the temporal bone, and the bullas were therefore easily detached from the bone. Afterwards, it was possible to identify the stapedial artery, RW, cochlea and ossicles (Figure 3b). The cochlea was then isolated carefully by detaching it from the suture lines attached to the adjacent bones. After fixation and decalcification of the cochlea with a 4% paraformaldehyde solution (0.1 M PBS, pH 7.4 for 8 h) and 0.1 M 10% EDTA solution (20 days), specimens were aligned on a cryomatrix for sectioning and histopathological analyses (Figure 4a). Alignment was adjusted so that the axial line between the apex of the cochlea and the cochlear nerve was parallel to the cutting plane. The transparency acquired after decalcification along with the stapedial artery was used as a guide for identifying the apex of the basal turn, and mid-modiolar sections were obtained (Figure 4b).

## RESULTS

The study group consisted of 40 adult female WARs, with a mean weight of 215 g. The mean time to reach the bulla and to achieve appropriate cochleostomy was 4.3 min (range 2-13 min) and 7.5 min (range 3.5-22 min), respectively. The width of the FN was approximately 1 mm (0.42-1.25 mm; mean: 0.84 mm). The mean area of the temporal bulla exposed was 14.3 mm2 (6.7-22.9 mm^2^). The size of the tympanic bulla was sufficient for easy identification and dissection. The mean area drilled on the bulla to expose the middle ear and the basal turn of cochlea was 5.1 mm2 (2.8-10 mm^2^). In all rats, the basal turn of the cochlea was easily identified. The stapedial artery lay nearly perpendicular to the course of the FN (88-93 °C).

Five rats had a contracted and hard bulla bone. In those dissections, the FN was re-routed to attain adequate exposure of the bulla. FN re-routing caused no significant weakness in the facial muscles. One rat had effusion in the middle ear, and the fluid was gently removed to identify the basal turn of the cochlea.

Surgical complications were encountered in three ears: an inadvertently large cochleostomy in two ears and massive arterial bleeding from the stapedial artery in one ear while performing the cochleostomy.

## DISCUSSION

As the number of studies focusing on inner ear drug therapies increases, the need to understand the anatomy of the experimental animal’s temporal bone inevitably emerges. However, inner ear injection studies require operating on a live animal and keeping the animal alive until the desired electrophysiological or histological effect is reached. Therefore, detailed knowledge of temporal bone anatomy and dissection techniques becomes crucial for such experiments. The retroauricular approach has been used for many years in rat temporal bone surgery. Many studies outline surgical techniques in a paragraph or two for cochlear gene or stem cell transfer experiments. However, we experienced great difficulty in repeating those steps and replicating those experiments in our own work. Therefore, we planned this paper to describe the surgical anatomy and access to the cochlea in detail with pictures and video presentations in order to produce useful information for other researchers, both experienced and inexperienced.

**Figure 5:**
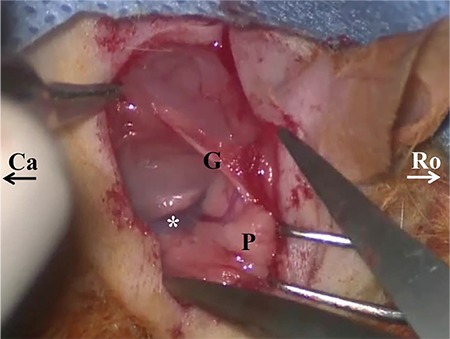
Ventral dissection to great auricular nerve exposed major blood vessels (asterisk). *Ro: rostral side, Ca: caudal side, G: great auricular nerve, P: parotid gland.*

The first difficulty in operating on a living rat is reaching the bulla in a timely manner without any nerve or great vessel injury. Therefore, completing the experiment safely and quickly enables the rapid recovery of the animal by using a lower dose of anaesthesia, resulting in fewer complications. We found that dissecting the trapezius muscle ventral to the great auricular nerve presents a risk to major blood vessels (Figure 5). Dorsal dissection and retraction of the trapezius muscle is a quicker and safer way to trace the FN and reach the bulla (Figure 1c, 1d, see video, supplemental digital content 3, demonstrating a. ventral dissection to the great auricular nerve and b. further dorsal dissection leading to the FN and otic bulla; G: great auricular nerve, P: parotid gland, asterisk: major blood vessels). We shortened our dissection time for the bulla to 2 min from 13 min (mean 4.3 min) and our cochleostomy time to 3.5 min from 22 min (mean 7.5 min) with this technique. There was no great vessel injury or nerve injury during dissection except one stapedial artery injury.

Retrograde tracing of the FN is the shortest and safest way to reach the bulla. Care must be taken to avoid injury during surgery because paralysed facial muscles may adversely affect the animal’s general health and its ability to feed. The FN of the rat is in a more superficial, anterior-rostral position and is less protected than the FN of cats and guinea pigs (11). Therefore, the FN may be injured during traction of the pinna or trapezius muscle or cut during resection of soft tissue. Additionally, the FN trunk was the upper drilling limit for the bulla, and care should be taken to prevent the FN from thermal or contact injury during drill-out. Re-routing of the FN in rostral and dorsal directions was required during the drill-out of poorly pneumatized bullas. Re-routing the FN or using a high-speed drill near the FN may cause some level of neuropraxis in otologic surgery. Interestingly, we encountered no FN weakness or injury due to retrograde tracing, bulla drilling or FN re-routing in our experiments. Electrophysiological and histopathological studies on rat FNs are suggested to clarify the differences between human and rodent FN physiology.

When performing cochleostomy, one must remember that the bone covering the basal turn of the cochlea is very thin and may easily be destroyed, possibly causing a perilymph gusher. Thus, it is safer to first thin the bone with a diamond burr and then manually make a hole with a 30-gauge perforator. The basal turn of the cochlea can be identified through a stereotactic microscope by noting the prominence of the turn ventrally to the stapedial artery. The RW was not fully visualized in every dissection. The stapedial artery limited direct access to the RW from the dissection plane in some instances. Further, dorsal-caudal drilling of the bulla needs to be done to directly access the RW.

In our study, there were three major complications. Two concerned oversized cochleostomy holes, which were then repaired with soft tissue grafts and fibrin sealant. The other complication was a massive bleed from the stapedial artery, which is worth stressing. The terminology is controversial because some reports name this structure an internal carotid artery while others describe it as a stapedial artery (6,12). The stapedial artery persists in some rodents, including rats, and forms one of the major blood supplies to the brain (6). Thus, preserving the artery during surgery is essential. However, in the case of bleeding, the stapedial artery can be cauterized for safe access to the RW (2).

While the cervical approach is the traditional way to access the tympanic bulla in rodents (1), the retroauricular approach provides simple and straightforward access to the cochlea. Also, it is a safe and reproducible method that can be used for experimental purposes. Our results suggest that the retroauricular approach provides acceptable access for inner ear drug delivery in WARs. Surgical steps, important landmarks and key points while performing temporal bone dissection in rats were discussed in this study. Possible complications and potential pitfalls of surgery were also evaluated and technical details were noted to guide researchers through the inner ear.
